# Trends and future directions in chronic rhinosinusitis with nasal polyps: A bibliometric analysis

**DOI:** 10.1016/j.bjorl.2025.101672

**Published:** 2025-07-03

**Authors:** Yeming Zhong, Xuan Wei, Caiyun Zou, Bo Qian, Hongbo Ji, Jinhe Guo, Zigang Che

**Affiliations:** a Southeast University, School of Medicine, Nanjing Tongren Hospital, Department of Radiology, Jiang Ning District, Nanjing, Jiangsu, China; b Southeast University, School of Medicine, Nanjing, Jiangsu, China

**Keywords:** Chronic rhinosinusitis with nasal polyps, Biologic therapy, Endoscopic surgery, Citation analysis, Bibliometric analysis

## Abstract

**Objectives:**

Chronic Rhinosinusitis with Nasal Polyps (CRSwNP) is a complex inflammatory condition that significantly impairs patients’ quality of life. Despite significant efforts to develop effective treatments, a comprehensive bibliometric analysis in this field is lacking. This study aims to identify key trends, influential publications, leading authors, and collaborative networks.

**Methods:**

A bibliometric analysis was conducted on October 16, 2024, examining publications in the Web of Science Core Collection (WoSCC) database from 2004 to 2024. Data visualization and interpretation were performed using VOSviewer (version 1.6.20), CiteSpace (version 6.3.R1), and R 4.3.3.

**Results:**

The analysis included 1,235 publications from 5,825 authors across 4,723 institutions in 222 countries. The United States led in publication volume, followed by China and Italy. Major institutional contributors included Capital Medical University and Sanofi. The International Forum of Allergy & Rhinology published the most articles, while the Journal of Allergy and Clinical Immunology had the highest citation count. Key authors identified were Bachert Claus and Gevaert Philippe. Keyword analysis revealed “asthma”, “endoscopic sinus surgery”, and “inflammation” as dominant themes, with burst analysis indicating growing interest in “humanization”, “type 2 inflammation”, “risk”, “biologics”, and “safety”.

**Conclusion:**

This bibliometric analysis underscores an increasing focus on biologic therapies, inflammation management, and endoscopic sinus surgery in CRSwNP research. Future investigations are likely to prioritize the long-term safety and cost-effectiveness of biologics, alongside advancements in precision medicine and surgical techniques.

**Level of evidence:**

Level 1.

## Highlights

• CRSwNP significantly impairs patients' quality of life.
• Study analyzes publications from 2004 to 2024.
• United States leads in publication volume.
• Key themes: inflammation, biologics, and surgery.
• Future focus on safety and precision medicine.

## Introduction

Chronic Rhinosinusitis with Nasal Polyps (CRSwNP) is a prevalent and debilitating inflammatory condition of the sinonasal mucosa, primarily driven by multifactorial etiology including genetic predispositions, environmental factors, and immune dysregulation.^1^ This results in symptoms such as nasal obstruction, loss of smell, facial pain, and a significant reduction in quality of life.^2^ The global prevalence of CRSwNP varies, affecting approximately 2%–4% of the population, with higher incidence rates reported in countries like China, where environmental factors and genetic predispositions may play a role.^1^, ^3^ Managing CRSwNP presents a significant burden on healthcare systems due to its chronic and multifactorial nature.^4^

In the past two decades, treatment strategies for CRSwNP have evolved significantly, encompassing medical therapies and surgical interventions. Corticosteroids, both topical and systemic, are widely used for their anti-inflammatory effects but carry risks, particularly with long-term use.^2^, ^4^ Endoscopic Sinus Surgery (ESS) is often recommended for patients unresponsive to medical therapy; however, it has its own limitations, including the potential for postoperative complications and polyp recurrence.^5^ While ESS can provide symptom relief and improve quality of life, outcomes can vary, and the risk of recurrence remains a concern.^6^ Recently, biologic therapies have emerged as promising alternatives, offering targeted treatments with fewer systemic side effects, yet they remain costly and require long-term administration.^7^ These varied treatments outcomes underscore the need for ongoing research into optimizing CRSwNP therapies to provide clinicians with safer and more effective options for improving patient outcomes.

Bibliometric analysis is an essential tool for systematically assessing research trends, citation patterns, and collaborative networks in a given field.^8^ High-impact bibliometric studies have demonstrated its utility in highlighting key developments and influential contributions across medical disciplines.^9^ In the context of CRSwNP treatment, bibliometric analysis allows us to identify not only the most influential studies and researchers but also emerging trends in therapeutic approaches, such as the increased adoption of biologics and advancements in surgical techniques.^10^ This approach is crucial for understanding the evolving landscape of CRSwNP management and ensuring that future research efforts are directed toward the most promising and impactful areas.^11^ This study conducted a bibliometric analysis of CRSwNP treatment research from 2004 to 2024, focusing on citation patterns, co-authorship networks, and keyword trends. By identifying key trends and influential contributions, this analysis aims to guide future research directions and inform clinical practice to improve patient outcomes in CRSwNP management.

## Methods

### Search strategies and data collection

A thorough literature search was carried out using the Web of Science Core Collection (WoSCC) to identify studies related to CRSwNP treatment published between January 1, 2004, and October 16, 2024. The search terms used were: TS = ((“Chronic Rhinosinusitis with Nasal Polyps” OR CRSwNP)) AND TS = (treatment OR therapy OR therapeutics OR management OR intervention OR “pharmacological treatment” OR “surgical treatment” OR “medical treatment” OR “endoscopic sinus surgery” OR “ESS” OR “nasal steroid” OR “biologics” OR “systemic corticosteroids” OR “antibiotics” OR “leukotriene receptor antagonists” OR “nasal irrigation” OR “saline irrigation” OR “anti-IL5 therapy” OR dupilumab OR omalizumab OR mepolizumab OR “monoclonal antibodies”). The search was restricted to articles in English, and to avoid potential biases from database updates, it was conducted on October 16, 2024. The data were formatted as text for subsequent bibliometric analysis. The information extracted included publications and citation counts, article titles, authors, institutional affiliations, countries/regions, keywords, and journal names.

### Statistical analysis

Key bibliometric indicators such as annual publication numbers, citation counts, average citation rates, journal Impact Factors (IF), country/region contributions, institutional affiliations, and author metrics were calculated using Microsoft Excel. Excel allowed for efficient data organization and analysis.

For the visual representation of the data, three bibliometric tools: VOSviewer (version 1.6.20), CiteSpace (version 6.3.R1), and the *R* (version 4.3.3) bibliometric platform (https://bibliometric.com/) were employed. VOSviewer was used to create visual maps of institutional and author collaborations, co-authorship, citations, and co-citations,^12^ enabling us to explore collaborative networks and relationships between key contributors. Additionally, we used VOSviewer for keyword co-occurrence analysis and CiteSpace for identifying emerging trends and keyword bursts. CiteSpace was configured to analyze data from January 2004 to June 2024, using annual time slices. For keyword analysis, we applied a threshold of the top five keywords per time slice, and used pathfinder and network pruning methods to refine the visualizations, ultimately producing a keyword timeline map for CRSwNP treatment research. In the visualizations, node sizes represent publication counts, line thickness reflects link strength, and node colors indicate different clusters or time periods. The H-index values were sourced from the WoSCC, was employed to evaluate the academic impact of researchers and journals, serving as a crucial metric for assessing scientific contributions.^13^

## Results

### Overall review

**Fig. 1:**
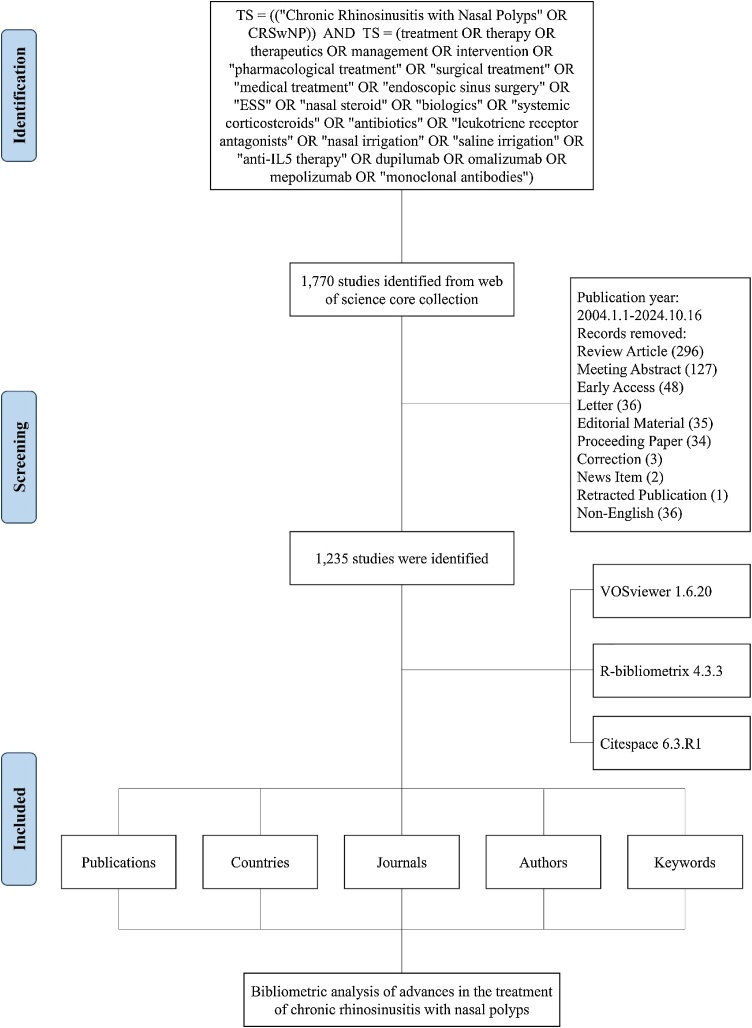
Flowchart depicting the literature screening process for studies on CRSwNP treatment.

**Fig. 2:**
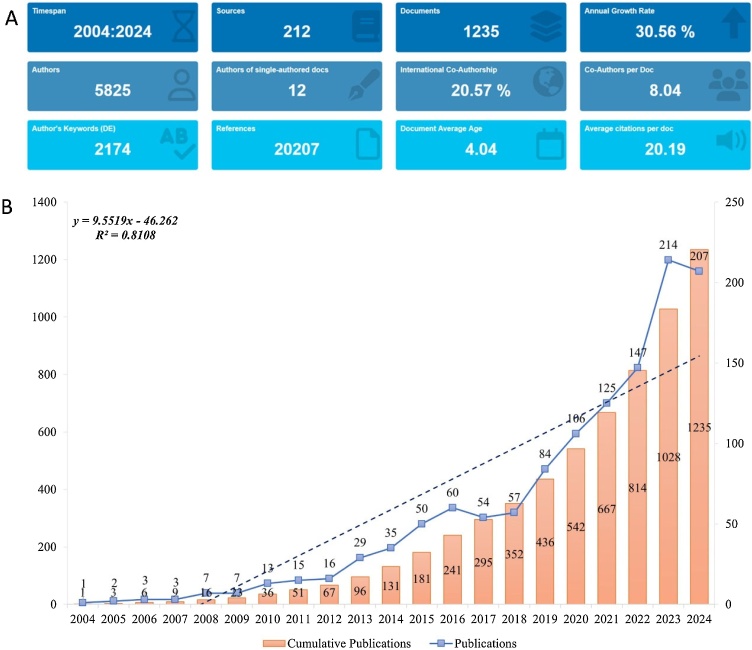
Overview and Trends in Publications on CRSwNP treatment from 2004 to 2024. (A) Number of Publications on CRSwNP treatment. (B) Annual Number of Publications on CRSwNP treatment.

Ultimately, 1235 publications met the inclusion criteria for this study, with the data screening process depicted in Fig. 1. This study analyzed contributions from 5,825 authors across 4,723 institutions and 222 countries, collectively citing 20,207 references between 2004 and 2024 (Fig. 2A). The number of annual publications has shown a clear upward trend, reflecting the growing interest and research efforts in CRSwNP treatment over the past two decades (Fig. 2B). The annual average output was approximately 62 papers, with 2023 achieving the highest publication count (n = 214).

### Analysis of countries and institutions

**Fig. 3:**
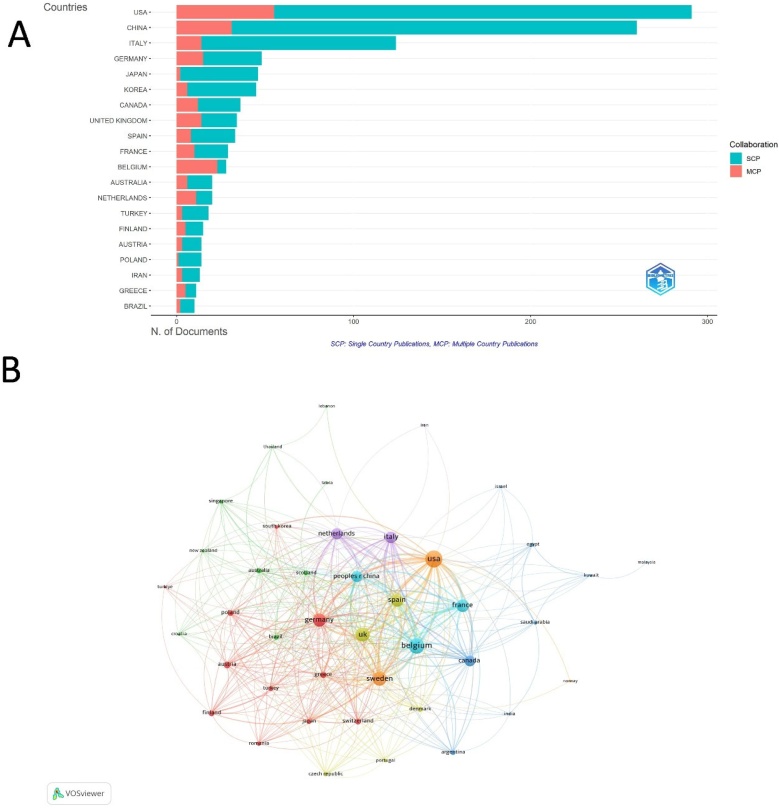
Global Research Distribution and Collaboration in CRSwNP Treatment. (A) Map displaying the distribution of publications by corresponding authors’ countries, differentiating between Single Country Publications (SCP) and Multi-Country Publications (MCP). (B) Collaboration map indicating inter-country cooperation, with node size representing publication count and link thickness reflecting co-authorship strength.

Among the leading countries contributing to publications on CRSwNP treatment, the United States ranked first with 291 articles, accounting for the highest number of publications. It was followed by China with 260 articles and Italy with 124 publications. In terms of research collaboration strength, Belgium stood out with the highest ratio of Multiple-Country Publications (MCP), with 82.1%, followed by the Netherlands (55%) and the Greece (45.5%). In regard to Total Citations (TC), United States ranked first (8,606), followed by China (3,332) and Belgium (2,932) (Supplementary Table 1 and Fig. 3A). Among the 45 countries involved in international collaborations with a minimum of 3 articles, the USA has the highest number of collaborations with other countries (total link strength = 454), followed by Belgium (372) and Sweden (319) (Fig. 3B).

**Fig. 4:**
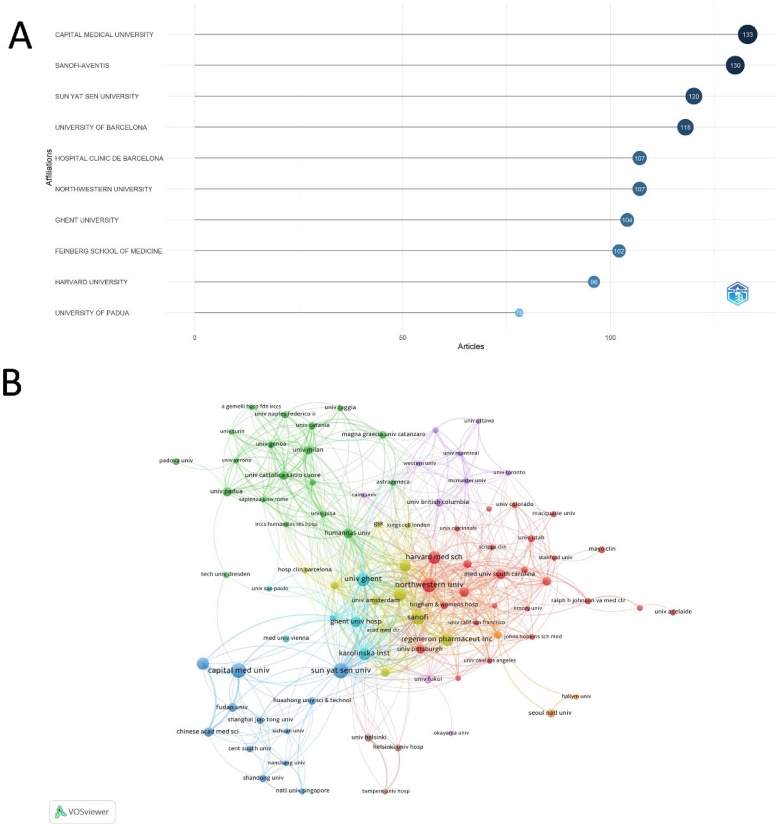
Institutional Contributions and Collaborations in CRSwNP Treatment. (A) Chart of the top 10 institutions by article count and ranking, with circle size indicating publication volume and color depth denoting rank. (B) Institutional collaboration map, where nodes are institutions sized by publication count, and links represent co-authorship strength, colored to differentiate research clusters.

The Capital Medical University in China ranked first with 133 publications. Sanofi-Aventis followed closely with 130 publications, while Sun Yat Sen University and the University of Barcelona contributed 120 and 118 articles, respectively. Other prominent institutions included Hospital Clinic de Barcelona and Northwestern University, both with 107 publications each (Fig. 4A). In terms of research collaboration, Among the 92 institutions involved in international collaborations with a minimum of 8 articles, Sanofi demonstrated the highest number of international partnerships, with 223 collaborations, followed by Ghent University (216 collaborations) and Regeneron Pharmaceuticals (199 collaborations) (Fig. 4B).

### Analysis of prominent journals and collaborations

The International Forum of Allergy & Rhinology ranked first in publication volume, with 145 articles and 2,556 TCs, underscoring its significant impact. The American Journal of Rhinology & Allergy followed with 97 articles, but ranked sixth in TCs (1,538). *Rhinology,* with 82 articles and 2,403 TCs, secured the third spot in both Total Publications (TP). In terms of citation impact, the Journal of Allergy and Clinical Immunology had the highest TCs (4,891) despite only 32 publications, highlighting its significant influence in the field (Supplementary Table 2).

**Fig. 5:**
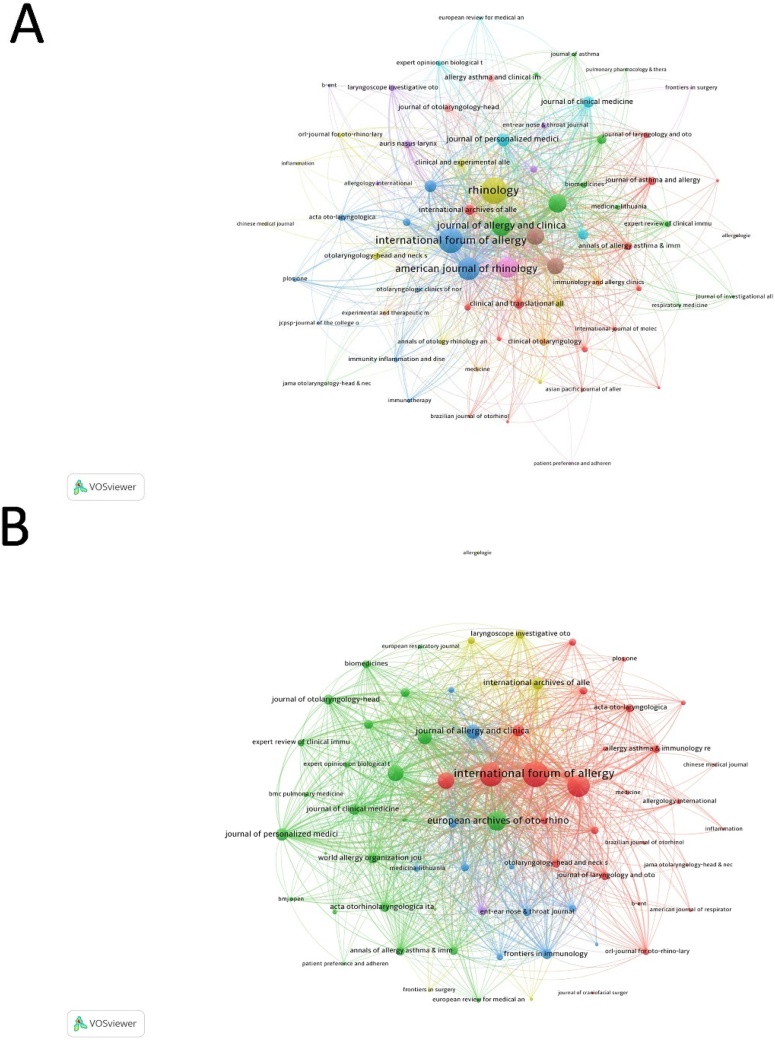
Analysis of Journal Networks in CRSwNP Treatment Research. (A) Co-occurrence networks show thematic connections between journals based on citation patterns within articles. (B) Coupling networks illustrate intellectual linkages between journals through commonly cited references.

The co-occurrence networks of journals contained 70 with at least 3 occurrences. The three key journals with the highest total link strength in Co-occurrence Networks were the rhinology (972), international forum of Allergy & Rhinology (906), and American Journal of Rhinology & Allergy (702) (Fig. 5A). The coupling networks showed that the three key journals with the highest total link strength in Co-occurrence Networks were international forum of Allergy & Rhinology (75932), Rhinology (61356), and American Journal of Rhinology & Allergy (57924) (Fig. 5B).

### Analysis of authors

**Fig. 6:**
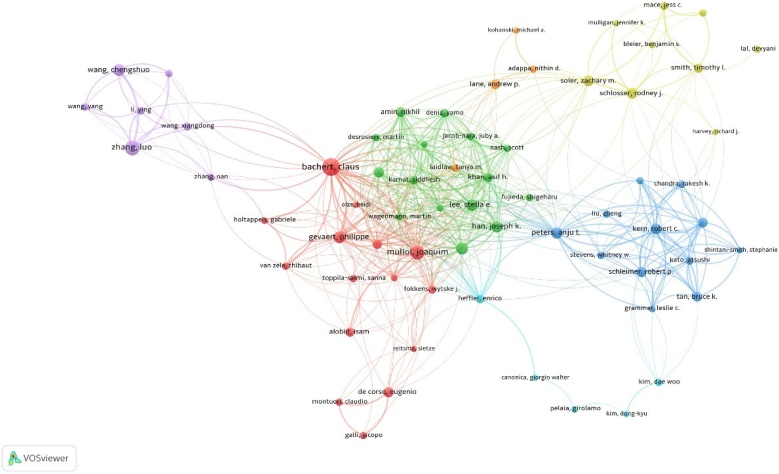
Author collaboration network in CRSwNP treatment.

Our analysis identified 20 high-impact authors in CRSwNP research based on their H-index, TP, and TC (Supplementary Table 3). Bachert Claus ranked first with an H-index of 36-, 68 publications, and 5,435 citations, making him the most influential author in the field. Luo Zhang followed with 46 publications, while Gevaert Philippe and Joaquim Mullol ranked second and third in total citations (2,934 and 2,903, respectively). Among the 76 authors involved in international collaborations with a minimum of 9 articles, Bachert Claus has the highest number of collaborations with other countries (287), followed by Peters Anju T. (154) and Mullol Joaquim (143) (Fig. 6).

### Analysis of papers

The most cited publication was the 2019 study, “Efficacy and safety of dupilumab in patients with severe chronic rhinosinusitis with nasal polyps (LIBERTY NP SINUS-24 and LIBERTY NP SINUS-52): results from two multicenter, randomized, double-blind, placebo-controlled, parallel-group phase 3 trials” published in The Lancet (IF = 168.9), which has garnered 772 citations.^14^ This study was instrumental in demonstrating the efficacy of dupilumab, a biologic therapy, in reducing polyp size and improving symptoms. Another highly cited paper was the 2015 study, “Novel scoring system and algorithm for classifying chronic rhinosinusitis: the JESREC Study” published in the *Allergy* (IF = 12.6) with 436 citations.^15^ The third-ranking article, “Efficacy and safety of omalizumab in nasal polyposis: 2 randomized phase 3 trials” published in the Journal of Allergy and Clinical Immunology (IF = 14.2) with 366 citations.^10^

### Analysis of keywords and brust keywords

**Fig. 7:**
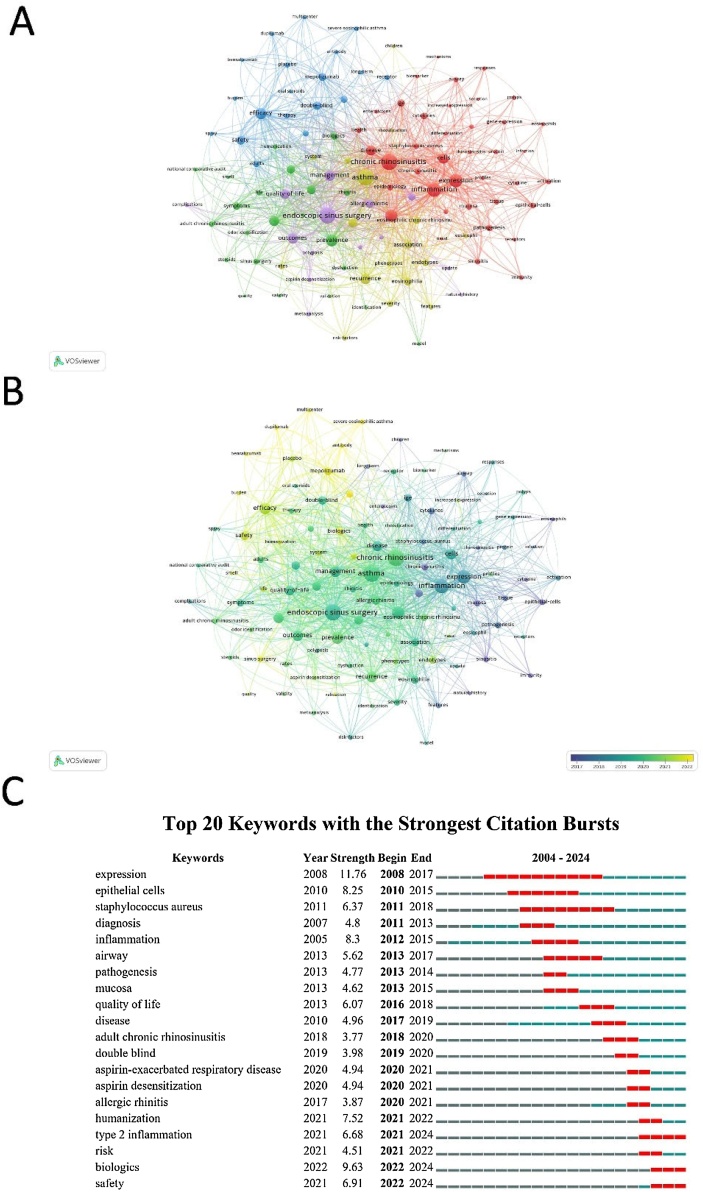
Keyword Co-occurrence Network and Citation Burst Analysis in CRSwNP Treatment. (A) Keyword co-occurrence clustering analysis. (B) Keyword co-occurrence network analysis. (C) Citation Burst Analysis.

Our keyword analysis identified 111 recurring terms with a minimum of 11 occurrences, offering insights into the main research themes in CRSwNP treatment. “Chronic rhinosinusitis” (258 occurrences) was the most frequent keyword, followed by “Asthma” (211 occurrences) and “Endoscopic sinus surgery” (205 occurrences). Other key terms included “Inflammation” (155 occurrences) and “Expression” (161 occurrences). The keyword clustering provides a visual representation of the different thematic areas in CRSwNP research. The red cluster centers on inflammation and related molecular mechanisms such as “expression” and “cells,” highlighting the biological and immunological aspects of CRSwNP. The yellow cluster is focused on surgical interventions, with keywords like “endoscopic sinus surgery”, “recurrence”, and “outcomes”, indicating research on treatment effectiveness and long-term patient management. The green cluster is primarily concerned with asthma and its comorbidities, reflecting the connection between asthma and CRSwNP in clinical research. The blue cluster emphasizes efficacy and management, focusing on treatment outcomes, including biologics and other medical interventions (Fig. 7A).

The co-occurrence network (Fig. 7B**)** revealed strong associations between keywords, with “Asthma”, “endoscopic sinus surgery” and “inflammation” showing the highest total link strengths (817, 775 and 571, respectively). Additionally, terms like “Efficacy” (94 occurrences) and “Safety” (56 occurrences) are gaining attention, especially in relation to treatment outcomes and patient management strategies.

Burst keyword analysis (Fig. 7C) highlighted emerging terms that have gained prominence in recent years. “Biologics” (2022–2024), “Type 2 inflammation” (2021–2024), and “Safety” (2022–2024) show strong citation bursts, reflecting growing interest in novel therapies for CRSwNP. While terms such as “Humanization” and “Risk” have become more relevant since 2021, indicating a shift towards precision medicine and risk management in treatment.

## Discussion

This bibliometric analysis of CRSwNP treatment research from 2004 to 2024 shows a significant increase in research output over the years, with the United States, China, and Italy emerging as the leading contributors. Keyword analysis revealed “asthma”, “endoscopic sinus surgery” and “inflammation” as frequent terms, with a clear shift towards biologic therapies, gaining prominence in recent years. These findings emphasize the growing global focus on CRSwNP treatment, the critical role of international collaborations, and the increasing research attention on biologics and precision medicine.

The International Forum of Allergy & Rhinology emerged as the leading journal in terms of publication volume, with 145 articles contributing significantly to the dissemination of CRSwNP research. However, the Journal of Allergy and Clinical Immunology stood out as the most influential journal, evidenced by its highest TC. This highlights the journal’s pivotal role in shaping the direction of CRSwNP research, particularly in the areas of immunology and biologic therapies, which have gained prominence in recent years.^16^, ^17^ Additionally, Rhinology and the American Journal of Rhinology & Allergy also played crucial roles, both in advancing surgical techniques and in exploring the immunological aspects of CRSwNP management.^4^ The strong citation impact of these journals illustrates the field’s growing emphasis on understanding the immune mechanisms underlying CRSwNP and the development of targeted, biologic-based treatments.^18^ Given the increasing focus on precision medicine and biologic therapies, future research should continue to integrate interdisciplinary approaches that combine immunology, pharmacology, and clinical applications to further optimize treatment strategies for CRSwNP.^19^

The United States, China, and Italy emerged as the top contributors to CRSwNP research, with the United States leading in publication volume and demonstrating the highest level of international collaborations, particularly with European countries such as Belgium and the Netherlands. This extensive collaboration network underscores the United States’ role in fostering global knowledge exchange in the field of CRSwNP, contributing to the advancement of both biologic therapies and surgical techniques. China ranked second in publication volume and, despite having fewer international collaborations, demonstrated a growing impact in the field, driven by its increasing research output and significant contributions from institutions such as Capital Medical University.^20^ Italy also played a key role in CRSwNP research, particularly through collaborations with other European institutions. The prominence of Capital Medical University and other Chinese institutions reflects the country’s substantial investment in respiratory and immunological research, likely motivated by the high prevalence of CRSwNP due to environmental factors and the large population base.^20^ European institutions, such as Ghent University and the University of Barcelona, were recognized for their significant contributions, particularly in the development of biologic therapies, positioning them as leaders in targeted CRSwNP treatments. The involvement of industry-leading institutions like Sanofi-Aventis and Regeneron Pharmaceuticals further highlights the importance of private sector collaborations in advancing biologic research. Moving forward, it will be essential to expand international collaborations, integrating diverse perspectives and leveraging global expertise to further the development of novel biologic therapies and precision medicine approaches to CRSwNP management.^2^

The most influential authors in the field, such as Bachert Claus and Mullol Joaquim, have significantly contributed to advancing biologic therapies and understanding the immunopathology of CRSwNP.^14^, ^21^ Bachert’s work on anti-IL-5 treatments and Mullol’s contributions to endoscopic surgery innovations have laid the groundwork for many clinical advances.^22^, ^23^ Through their extensive publications and high citation counts, these authors have shaped the current treatment paradigms. Their research often focuses on the intersection of immunology and clinical practice, offering insights into how biologic therapies can be integrated into standard care. Their contributions highlight the importance of targeted therapies in managing CRSwNP, particularly in cases where conventional treatments have failed. Future studies should continue to build on their work, particularly in long-term safety evaluations of biologics and the exploration of new therapeutic targets.^24^

The keyword analysis revealed that “asthma”, “endoscopic sinus surgery” and “inflammation” remain central to CRSwNP research, underscoring the persistent focus on understanding the disease’s interconnected conditions and treatment approaches.^25^ The growing prevalence of keywords such as “biologics” in recent years highlights the increasing emphasis on biologic therapies as a major area of research interest.^26^, ^27^ This shift reflects a broader trend in the field toward precision medicine, where targeted therapies are being explored to address the underlying inflammatory processes in CRSwNP. The consistent appearance of keywords related to “inflammation” and “endoscopic sinus surgery” suggests that while biologics are becoming more prominent, the role of surgery in managing CRSwNP continues to be an important research area. Efforts to refine surgical techniques and improve patient outcomes remain a high priority, particularly in cases where medical management alone is insufficient.^28^ Additionally, terms like “expression” and “cells” indicate ongoing advancements in molecular and genetic research, pointing to the development of humanized antibodies and personalized therapies, which are expected to play a significant role in future CRSwNP treatment strategies.^29^

The timeline of keyword bursts further confirms the rise of biologics as a research hotspot, particularly since 2017, corresponding with the introduction of drugs like dupilumab.^30^ The recent prominence of keywords such as “safety” and “efficacy” reflects increasing concern about the long-term effects of biologics, suggesting that future research should prioritize evaluating both the safety profile and the cost-effectiveness of these treatments, especially in diverse patient populations.^14^ To ensure that biologic therapies become more accessible and beneficial to a broader range of patients, future studies should focus on identifying new molecular targets and developing more affordable biologic options.^31^, ^32^

### Future research directions and limitations

While this study provides a detailed analysis of the CRSwNP research landscape, it is essential to address the limitations of bibliometric analysis. Firstly, the analysis was limited to English-language publications, which may have excluded significant contributions from non-English-speaking countries. Secondly, citation counts, while useful, do not always reflect the clinical impact or quality of studies, particularly for newer publications that have not had sufficient time to accumulate citations. To gain a more comprehensive understanding, future studies should incorporate a wider range of databases and include grey literature such as conference proceedings and government reports. Looking ahead, research should focus on further refining biologic therapies, exploring new molecular targets, and improving the accessibility of these treatments.^33^ Long-term safety studies, cost-effectiveness analyses, and personalized medicine approaches will be critical in shaping the future of CRSwNP management.^34^ Moreover, interdisciplinary collaborations combining immunology, pharmacology, and clinical researches will be necessary to overcome the current challenges and develop more effective treatment strategies.

## Conclusion

This bibliometric analysis provides valuable insights into the evolving landscape of CRSwNP treatment research. The focus on biologic therapies, the refinement of surgical techniques, and the growing international collaborations have greatly contributed to advancements in this field. Future research should continue to explore the long-term efficacy and safety of biologics, identify new therapeutic targets, and foster global collaborations to further enhance treatment outcomes for CRSwNP patients.

## ORCID ID

Yeming Zhong: 0000-0002-9892-3625

Xuan Wei: 0009-0001-9164-9491

Bo Qian: 0009-0009-7336-2807

Hongbo Ji: 0009-0005-5458-0746

Caiyun Zou: 0000-0002-0611-2981

Jinhe Guo: 0000-0002-4620-2930

Zigang Che: 0000-0003-2039-7925

## CRediT authorship contribution statement

Conception and design: Jinhe Guo, Zigang Che.

Administrative support: Caiyun Zou.

Data analysis and interpretation: Yeming Zhong, Xuan Wei.

Manuscript writing: All authors.

Final approval of manuscript: All authors.

## Consent for publication

Not applicable.

## Ethics approval and consent to participate

Not applicable.

## Funding

The research was funded by The Nanjing Medical Science and technique Development Foundation, Contract grant number: YKK23263.

## Availability of data and materials

All data generated or analyzed during this study are included in this published article.

## Declaration of competing interest

The authors declare no conflicts of interest.
