# Supplementary material for: Trends and future directions in chronic rhinosinusitis with nasal polyps: A bibliometric analysis
Source: Braz J Otorhinolaryngol. 2025 Jul 3;91(5):101672. doi: 10.1016/j.bjorl.2025.101672 (PMC12270626; doi:10.1016/j.bjorl.2025.101672)
Supplement: Supplementary file 1 [file mmc1.docx]

**BJORL-D-25-00096_ Supplementary Material**

**Supplementary Table 1** Publication and citation profiles of leading countries.

| **Country** | **Articles** | **Freq** | **SCP** | **MCP** | **MCP_ ratio** | **TP** | **TP_ rank** | **TC** | **TC_ rank** | **Average Citations** |
| --- | --- | --- | --- | --- | --- | --- | --- | --- | --- | --- |
| USA | 291 | 236 | 55 | 24 | 0.189 | 1259 | 1 | 8606 | 1 | 29.6 |
| China | 260 | 229 | 31 | 21 | 0.119 | 911 | 2 | 3332 | 2 | 12.8 |
| Italy | 124 | 110 | 14 | 10 | 0.113 | 806 | 3 | 1557 | 4 | 12.6 |
| Germany | 48 | 33 | 15 | 4 | 0.313 | 269 | 5 | 831 | 7 | 17.3 |
| Japan | 46 | 44 | 2 | 4 | 0.043 | 197 | 9 | 1443 | 5 | 31.4 |
| South Korea | 45 | 39 | 6 | 4 | 0.133 | 167 | 11 | 689 | 8 | 15.3 |
| Canada | 36 | 24 | 12 | 3 | 0.333 | 203 | 8 | 468 | 12 | 13 |
| United Kingdom | 34 | 20 | 14 | 3 | 0.412 | 223 | 6 | 601 | 9 | 17.7 |
| Spain | 33 | 25 | 8 | 3 | 0.242 | 303 | 4 | 522 | 10 | 15.8 |
| France | 29 | 19 | 10 | 2 | 0.345 | 207 | 7 | 326 | 14 | 11.2 |
| Belgium | 28 | 5 | 23 | 2 | 0.821 | 179 | 10 | 2932 | 3 | 104.7 |
| Australia | 20 | 14 | 6 | 2 | 0.300 | 99 | 13 | 518 | 11 | 25.9 |
| Netherlands | 20 | 9 | 11 | 2 | 0.550 | 70 | 15 | 931 | 6 | 46.5 |
| Turkey | 18 | 15 | 3 | 2 | 0.167 | 56 | 16 | 183 | 15 | 10.2 |
| Finland | 15 | 10 | 5 | 1 | 0.333 | 124 | 12 | 104 | 20 | 6.9 |
| Austria | 14 | 11 | 3 | 1 | 0.214 | 44 | 20 | 114 | 19 | 8.1 |
| Poland | 14 | 13 | 1 | 1 | 0.071 | 48 | 19 | 337 | 13 | 24.1 |
| Iran | 13 | 10 | 3 | 1 | 0.231 | 55 | 17 | 44 | 30 | 3.4 |
| Greece | 11 | 6 | 5 | 1 | 0.455 | 34 | 26 | 87 | 21 | 7.9 |
| Brazil | 10 | 8 | 2 | 1 | 0.200 | 39 | 24 | 180 | 16 | 18 |

Articles: Publications of Corresponding Authors only.

Freq, Frequence of Total Publications; SCP, Single Country Publications; MCP, Multiple Country Publications; MCP_Ratio: Proportion of Multiple Country Publications; TP, Total Publications; TP_rank, Rank of Total Publications; TC, Total Citations; TC_rank, Rank of Total Citations.

**Supplementary Table 2** Bibliometric indicators of high-impact journals.

| **Journal** | **h_index** | **g-index** | **m-index** | **TP** | **TP_ rank** | **TC** | **TC_ rank** | **PY_ start** | **IF_ 2023** | **JCR_ 2023** |
| --- | --- | --- | --- | --- | --- | --- | --- | --- | --- | --- |
| International Forum of Allergy & Rhinology | 34 | 50 | 2.429 | 145 | 1 | 2556 | 2 | 2011 | 7.2 | 1 |
| American Journal of Rhinology & Allergy | 24 | 41 | 1.500 | 97 | 2 | 1538 | 6 | 2009 | 2.5 | 1 |
| Rhinology | 23 | 39 | 1.353 | 82 | 3 | 2403 | 4 | 2008 | 4.8 | 1 |
| Journal of Allergy and Clinical Immunology | 22 | 32 | 1.048 | 32 | 6 | 4891 | 1 | 2004 | 11.4 | 1 |
| Laryngoscope | 19 | 40 | 0.950 | 53 | 5 | 2423 | 3 | 2005 | 2.2 | 1 |
| Allergy | 18 | 27 | 0.900 | 27 | 9 | 2212 | 5 | 2005 | 12.6 | 1 |
| Journal of Allergy and Clinical Immunology in Practice | 17 | 29 | 2.125 | 29 | 8 | 1086 | 8 | 2017 | 8.2 | 1 |
| European Archives of Otorhinolaryngology | 16 | 20 | 0.941 | 71 | 4 | 621 | 10 | 2008 | 1.9 | 2 |
| Allergy Asthma & Immunology Research | 10 | 12 | 0.833 | 12 | 23 | 230 | 27 | 2013 | 4.1 | 2 |
| American Journal of Otolaryngology | 10 | 16 | 1.000 | 29 | 7 | 224 | 28 | 2015 | 1.8 | 2 |
| Otolaryngology-Head and Neck Surgery | 10 | 17 | 0.556 | 17 | 13 | 1345 | 7 | 2007 | 2.6 | 1 |
| International Archives of Allergy and Immunology | 9 | 17 | 0.600 | 18 | 12 | 279 | 23 | 2010 | 2.5 | 3 |
| World Allergy Organization Journal | 9 | 13 | 1.500 | 16 | 15 | 148 | 47 | 2019 |  |  |
| Clinical and Experimental Allergy | 8 | 12 | 0.615 | 12 | 25 | 596 | 11 | 2012 | 6.3 | 1 |
| Immunology and Allergy Clinics of North America | 8 | 10 | 0.500 | 10 | 33 | 139 | 52 | 2009 | 2.7 | 3 |
| Journal of Personalized Medicine | 8 | 13 | 2.000 | 23 | 10 | 150 | 45 | 2021 | 3.0 | 1 |
| Acta Otolaryngologica | 7 | 12 | 0.500 | 13 | 19 | 292 | 22 | 2011 | 1.2 | 3 |
| Acta Otorhinolaryngologica Italica | 7 | 11 | 0.875 | 13 | 20 | 142 | 50 | 2017 | 2.1 | 2 |
| Auris Nasus Larynx | 7 | 13 | 0.500 | 13 | 21 | 218 | 31 | 2011 | 1.6 | 2 |
| Allergology International | 6 | 6 | 0.545 | 6 | 39 | 230 | 26 | 2014 | 6.2 | 1 |

Note(s): H_index: The H-index of the journal, which measures both the productivity and citation impact of the publications. IF, Impact Factor; indicating the average number of citations to recent articles published in the journal. JCR_Quartile: The quartile ranking of the journal in the Journal Citation Reports, indicating the journal's ranking relative to others in the same field (Q1: top 25%, Q2: 25%‒50%, Q3: 50%‒75%, Q4: bottom 25%). TP: Total Publications. TP_rank: Rank of Total Publications. TC: Total Citations. TC_rank: Rank of Total Citations. Average Citations: The average number of citations per publication. PY_start: Publication Year Start, indicating the year the journal started publication.

**Supplementary Table 3** Publication and citation profiles of high-impact authors.

| **Journal** | **h_index** | **g-index** | **m-index** | **PY_start** | **TP** | **TP_Frac** | **TP_rank** | **TC** | **TC_rank** |
| --- | --- | --- | --- | --- | --- | --- | --- | --- | --- |
| Bachert Claus | 36 | 68 | 1.90 | 2006 | 68 | 6.57 | 1 | 5435 | 1 |
| Gevaert Philippe | 24 | 33 | 1.26 | 2006 | 33 | 2.98 | 5 | 2934 | 2 |
| Mullol Joaquim | 22 | 42 | 1.38 | 2009 | 42 | 4.66 | 3 | 2903 | 3 |
| Zhang Luo | 19 | 33 | 1.73 | 2014 | 46 | 6.96 | 2 | 1153 | 14 |
| Hopkins Claire | 18 | 34 | 1.39 | 2012 | 34 | 4.51 | 4 | 2573 | 4 |
| Han Joseph K. | 17 | 30 | 1.00 | 2008 | 30 | 3.16 | 6 | 2095 | 6 |
| Schlosser Rodney J. | 16 | 26 | 0.94 | 2008 | 26 | 4.61 | 10 | 875 | 19 |
| Amin Nikhil | 15 | 20 | 2.50 | 2019 | 20 | 1.52 | 19 | 1747 | 8 |
| Lee Stella E. | 15 | 27 | 2.14 | 2018 | 27 | 3.17 | 8 | 2234 | 5 |
| Mannent Leda P. | 15 | 22 | 2.50 | 2019 | 22 | 1.60 | 14 | 1805 | 7 |
| Schleimer Robert P. | 15 | 22 | 0.88 | 2008 | 22 | 1.99 | 15 | 1589 | 10 |
| Wang Chengshuo | 15 | 29 | 1.50 | 2015 | 30 | 4.85 | 7 | 885 | 18 |
| Hellings Peter W. | 14 | 19 | 1.27 | 2014 | 19 | 1.26 | 23 | 1682 | 9 |
| Kern Robert C. | 14 | 24 | 1.00 | 2011 | 24 | 1.90 | 13 | 1383 | 11 |
| Peters Anju T. | 14 | 26 | 1.00 | 2011 | 26 | 2.55 | 9 | 1260 | 13 |
| Soler Zachary M. | 14 | 23 | 1.17 | 2013 | 25 | 4.34 | 12 | 571 | 21 |
| Lane Andrew P. | 13 | 19 | 0.68 | 2006 | 19 | 4.79 | 24 | 539 | 22 |
| Tan Bruce K. | 13 | 21 | 0.93 | 2011 | 21 | 2.18 | 18 | 1143 | 15 |
| Brescia Giuseppe | 12 | 19 | 1.20 | 2015 | 21 | 3.00 | 16 | 388 | 24 |
| Chandra Rakesh K. | 12 | 14 | 0.86 | 2011 | 14 | 2.41 | 33 | 898 | 17 |

Note(s): H_index: The h-index of the journal, which measures both the productivity and citation impact of the publications. g_index: The g-index of the journal, which gives more weight to highly-cited articles. m_index: The m-index of the journal, which is the h-index divided by the number of years since the first published paper. TP: Total Publications. TP_rank: Rank of Total Publications. TC: Total Citations. TC_rank: Rank of Total Citations. Average Citations: The average number of citations per publication. PY_start: Publication Year Start, indicating the year the journal started publication.
